# Immunodeficiency-Related Vaccine-Derived Poliovirus (iVDPV) Excretion in an Infant with Severe Combined Immune Deficiency with Spillover to a Parent

**DOI:** 10.3390/vaccines12070759

**Published:** 2024-07-09

**Authors:** Madhu Chhanda Mohanty, Geeta Govindaraj, Mohammad Ahmad, Swapnil Y. Varose, Manogat Tatkare, Anita Shete, Savita Yadav, Yash Joshi, Pragya Yadav, Deepa Sharma, Arun Kumar, Harish Verma, Ankita P. Patil, Athulya Edavazhipurath, Dhananjayan Dhanasooraj, Sheena Othayoth Kandy, Jayakrishnan Machinary Puthenpurayil, Krishnan Chakyar, Kesavan Melarcode Ramanan, Manisha Madkaikar

**Affiliations:** 1 Mumbai Unit, ICMR-National Institute of Virology, Haffkine Institute Campus, Acharya Donde Marg, Parel, Mumbai 400012, India; ercintswapnil@gmail.com (S.Y.V.); manogat96t@gmail.com (M.T.); patilankita0623@gmail.com (A.P.P.); 2 Government Medical College, Kozhikode 673008, India; geetakkumar@gmail.com (G.G.); athulyasurendran94@gmail.com (A.E.); dhanasooraj@gmail.com (D.D.); sheenadileep20@gmail.com (S.O.K.); mp.jayakrishnan@gmail.com (J.M.P.); drkrishnanc@gmail.com (K.C.); 3 Country Office, World Health Organization, New Delhi 110011, India; ahmadmoh@who.int (M.A.); sharmade@who.int (D.S.); akumar@who.int (A.K.); 4 Microbial Containment Laboratory, ICMR-National Institute of Virology, Pune 411021, India; anitaaich2008@gmail.com (A.S.); varshapatil111@yahoo.com (S.Y.); yashjos1401@gmail.com (Y.J.); hellopragya22@gmail.com (P.Y.); 5 World Health Organization, 1209 Geneva, Switzerland; vermah@who.int; 6 Aster MIMS, Kozhikode 673016, India; kesavan.mr7@gmail.com; 7 ICMR-National Institute of Immunohematology, Mumbai 400012, India; madkaikarmanisha@gmail.com

**Keywords:** iVDPV, primary immunodeficiency, inborn errors of immunity, poliovirus, polio eradication, end game strategy

## Abstract

In order to maintain the polio eradication status, it has become evident that the surveillance of cases with acute flaccid paralysis and of environmental samples must be urgently supplemented with the surveillance of poliovirus excretions among individuals with inborn errors of immunity (IEI). All children with IEI were screened for the excretion of poliovirus during a collaborative study conducted by the ICMR-National Institute of Virology, Mumbai Unit, ICMR-National Institute of Immunohaematology, and World Health Organization, India. A seven-month -old male baby who presented with persistent pneumonia and lymphopenia was found to have severe combined immune deficiency (SCID) due to a missense variant in the RAG1 gene. He had received OPV at birth and at 20 weeks. Four stool samples collected at 4 weekly intervals yielded iVDPV type 1. The child’s father, an asymptomatic 32-year-old male, was also found to be excreting iVDPV. A haploidentical hematopoietic stem cell transplant was performed, but the child succumbed due to severe myocarditis and pneumonia three weeks later. We report a rare case of transmission of iVDPV from an individual with IEI to a healthy household contact, demonstrating the threat of the spread of iVDPV from persons with IEI and the necessity to develop effective antivirals.

## 1. Introduction

Since the GPEI (Global Polio Eradication Initiative) was launched in 1988, there has been a more than 99.9% reduction in the number of cases, and wild poliovirus (WPV) types 2 and 3 have been eradicated [1]. The extensive use of OPV (oral poliovirus vaccine) in routine childhood immunization and in immunization campaigns and the introduction of IPV (inactivated poliovirus vaccine) are responsible for this phenomenal achievement. Polio eradication is at a critical juncture following the setbacks in the wake of the COVID-19 pandemic [2]. Wild poliovirus remains endemic in Afghanistan and Pakistan, while circulating vaccine-derived polioviruses (cVDPVs) in environmental samples, as well as from AFP cases, have been documented in several countries from where wild poliovirus has been eliminated, especially those with poor vaccine coverage [3,4]. Eradication of polio hinges primarily on rapid and reliable surveillance among individuals with acute flaccid paralysis in addition to environmental samples, including sewage and wastewater, and in individuals with inborn errors of immunity (IEI) [5]. Genetic drifts of 0.6% and more for type 2 and 1% or more for types 1 or 3 denote that they are vaccine-derived polioviruses (VDPVs) that have regained neurovirulence and contagiousness. VDPVs have the potential to lead to outbreaks and result in paralytic poliomyelitis. Outbreaks of cVDPVs have occurred in various parts of the world, including in several countries in Africa, Indonesia, and USA [3].

IEI, previously known as primary immune deficiency disorders, include a spectrum of inherited structural or functional defects of adaptive and/or innate immunity, which, although individually rare, result in significant mortality and morbidity when considered together [6]. These rare diseases are being diagnosed more frequently in the country nowadays, due to enhanced awareness among health professionals and improved availability of immunological and genomic tests [7]. Normal individuals can clear the polio vaccine virus from the gut within six to eight weeks. However, individuals with IEI are unable to mount an adequate immune response, resulting in a diminished ability to clear the vaccine virus, prolonged shedding, and the potential to result in outbreaks in unvaccinated or partially vaccinated populations [8,9,10]. India has been categorized as a high-risk country for the occurrence of iVDPV [5]. The strategies of The Global Polio Surveillance Action Plan 2022–2024 (GPSAP 2022–2024) outlines the establishment of iVDPV surveillance to sustain polio eradication as one of its key objectives, to enhance surveillance systems, and to contribute to the timely detection of polioviruses [11].

A collaborative study was conducted by the ICMR-National Institute of Virology, Mumbai Unit (ICMR-NIVMU) and ICMR-National Institute of Immunohematology, World Health Organization India, and six centers in Phase I, which was expanded to include 14 more centers in Phase II [12]. All cases with an underlying inborn error of immunity were screened for the excretion of poliovirus/non-polio enteroviruses. During the COVID-19 lockdowns and thereafter, the National Polio Surveillance Project rendered support for the collection of stool samples from patients’ homes. During the first phase, 157 patients were enrolled in the study, comprising 64 with predominantly antibody deficiency and 48 with combined immunodeficiency and other IEI categories. One iVDPV excreter was identified from a child with hyper IgM syndrome [13]. Government Medical College, Kozhikode, a large teaching hospital in North Kerala, participated in the study from its inception. In Phase II of the study, iVDPV was identified on cell culture from four consecutive stool samples of a male infant with SCID. The same virus was also identified in stool samples of the child’s father. Here, we describe the virological and molecular characterization of the iVDPVs isolated from the IEI patient and his father and the immunological parameters associated with the patient.

## 2. Materials and Methods

In Phase II of the iVDPV study, patients with suspected IEI, based on clinical manifestations [ESID (European Society for Immunodeficiency) warning signs], were investigated for IEI. Stool samples were collected from diagnosed IEI patients who routinely visited the hospital/clinic for checkup and intravenous immunoglobulin (IVIg) treatment. The samples were collected either at the hospital or through the National Polio Surveillance Project, NPSP (WHO) if the patient was not visiting the hospital. The samples collected at each collaborating institute were transported to ICMR-NIVMU for further testing, which was conducted as per the methods mentioned below. The patients found positive for polio/ non-polio enteroviruses were subsequently followed-up until two subsequent samples became negative.

### 2.1. Immunophenotyping

Ethical clearance was obtained from the Ethics Committee of the ICMR-National Institute of Virology, Pune and Government Medical College, Kozhikode, Kerala, India. Blood samples were collected for the diagnosis of IEI. The immunophenotyping was conducted at Government Medical College, Kozhikode, India.

Flow cytometry was performed to analyze the lymphocyte subsets using the BD Multitest 6-color TBNK reagent in Beckman Coulter, Navious EX Flowcytometer and Kaluza Software V 2.1 [13]. The cell surface markers were evaluated using the following reagents: T cells (anti-CD3 PerCP-Cy5), B cells (anti-CD19 APC), monocytes (anti-CD14), and anti-HLA-DR (anti-HLA DR FITC). Naive T cell subsets on CD4 and CD8 cells were measured by anti-CD45RA-PE and anti-CD62L-APC) (BD Biosciences, San Jose, CA, USA). RAG-1 gene mutation was confirmed by NGS.

### 2.2. Stool Sample Collection, Processing, and Enterovirus Isolation

Processing of stool samples, culture, isolation, and characterization of enteroviruses were performed using the WHO laboratory manual, as described earlier [14], and iVDPV surveillance protocol [15]. Stool extract was inoculated in RD cells (human rhabdomyosarcoma) and L20B cells (transgenic mouse cell line expressing poliovirus receptor) for culture and the occurrence of the cytopathic effect (CPE). Intratypic differentiation of the isolates was analyzed by real-time PCR. Further characterization of the isolates was performed by VP1 (906 nt) region sequencing.

### 2.3. RT-PCR and Sanger Sequencing

For Sanger sequencing, viral RNA was extracted from the freeze–thaw lysate of the infected RD cell culture supernatant using the QIAamp Viral RNA Mini kit (manufactured by QIAGEN, Chatsworth, CA, USA). For the amplification of the VP1 region (906 nt), reverse transcriptase PCR was conducted in a single tube using reverse primer Q8 and forward primer Y7R, following a previously described protocol [16]. The PCR amplicons of the desired lengths were purified from agarose gel slices using the QIAquick Gel Extraction kit (QIAGEN, Chatsworth, CA, USA). Subsequently, sequencing was performed using the Big Dye Terminator v3.1 Cycle Sequencing Kit (manufactured by Applied Biosystems, Foster City, CA, USA) according to the manufacturer’s instructions (available at http://www.appliedbiosystems.com accessed on 1 August 2022). The resulting sequences were resolved on an ABI 3130xl Genetic Analyzer (Applied Biosystems, Foster City, CA, USA) and edited using Sequencher v4.10.1 software (developed by Gene Codes, Ann Arbor, MI, USA).

### 2.4. Comparative Analysis of Nucleotide and Amino Acid Sequences

The poliovirus reference strains retrieved from GenBank were employed for aligning the VP1 region and conducting sequence analysis. The alignment was performed using the CLUSTAL W program, which is embedded in MEGA7 (available at http://www.megasoftware.net accessed on 16 April 2024). Subsequently, both nucleotide and amino acid sequences were analyzed and compared pairwise.

### 2.5. NGS

Briefly, the ribosomal RNA depletion was performed using the NEBNext rRNA depletion kit (New England Biolabs, Ipswich, MA, USA). RNA NGS library preparation was performed using the Illumina TrueSeq stranded mRNA library preparation kit (Illumina, San Diego, CA, USA), which involves fragmentation and amplification. The amplified RNA libraries were quantified using Qubit quantification (Qubit dsDNA high-sensitivity assay kit, Thermo Fisher Scientific, Waltham, MA, USA). The quantified libraries were normalized and loaded on the Illumina sequencing MiniSeq platform. The raw Fastq files were mapped with the poliovirus reference sequence (Accession ID AY184219) using CLC genomics version 23.0.4. to retrieve the complete genome of the virus. Further, the sequences were analyzed on BLAST to explore the sequence similarity. Mapping with the PV1 Sabin strain AY184219, we could retrieve 99.99%, 99.97%, 99.83%, and 99.87% genome from all four samples, respectively. Coverage above 10X and a mutation frequency above 50% were considered for generating variant calling files (VCF). References of Christodoulou C et al. and Minor PD et al. were used to identify neurovirulent mutations, as well as nucleotide changes from the human poliovirus strain Sabin-1 to the Mahoney strain [17,18].

### 2.6. Poliovirus Neutralizing Antibody Titer

Poliovirus-neutralizing antibody titers were quantified using standard micro-neutralization assays. The challenge virus consisted of Sabin poliovirus vaccine strains obtained from NIBSC, UK, while HEp-2 Cincinnati cells served as the cell substrate. Serial 2-fold dilutions (ranging from 1:8 to 1:1024) of the serum samples were employed.

### 2.7. Cytokine Assay

Multiplex cytokine analysis kits from Merck (Milliplex) were utilized for cytokine assessments, and the tests were carried out in duplicate following the manufacturer’s instructions. Thirteen cytokines/chemokines were examined, encompassing pro-inflammatory ones (such as IL-1β, IL-6, IL-8, and TNF-α) and Th1 cytokines (including IFN-α2, IFNγ, and TNFα), as well as associated cytokines and chemokines (MCP-1, MIP-1α, MIP-1β, IP-10, FRACTALKINE, MIG, and VEGF-α). These analyses were conducted on serum samples using the Luminex-100 system, Version 1.7, from Luminex in Austin, TX, USA. The data were subsequently processed and analyzed with the MasterPlex QT 1.0 system from MiraiBio in Alameda, CA, USA using a five-parameter regression formula to calculate sample concentrations based on standard curves.

## 3. Results

### 3.1. Clinical Suspicion and Diagnosis of Inborn Errors of Immunity (IEI)

A 7-month-old male child from Malappuram District, Kerala, the third child of non-consanguineous parents, presented with persistent pneumonia for the last two months. His growth parameters and developmental milestones were normal. The child received two doses of OPV (birth dose and at 20 weeks) (Table 1). Investigations revealed lymphopenia, and he was suspected to have SCID. Nephelometric assay showed reduced levels of IgG (0.35 g/L) and IgM (0.16 g/L), with normal levels of IgA (0.26 g/L) and IgE (17.1 IU/mL).

**Figure 1:**
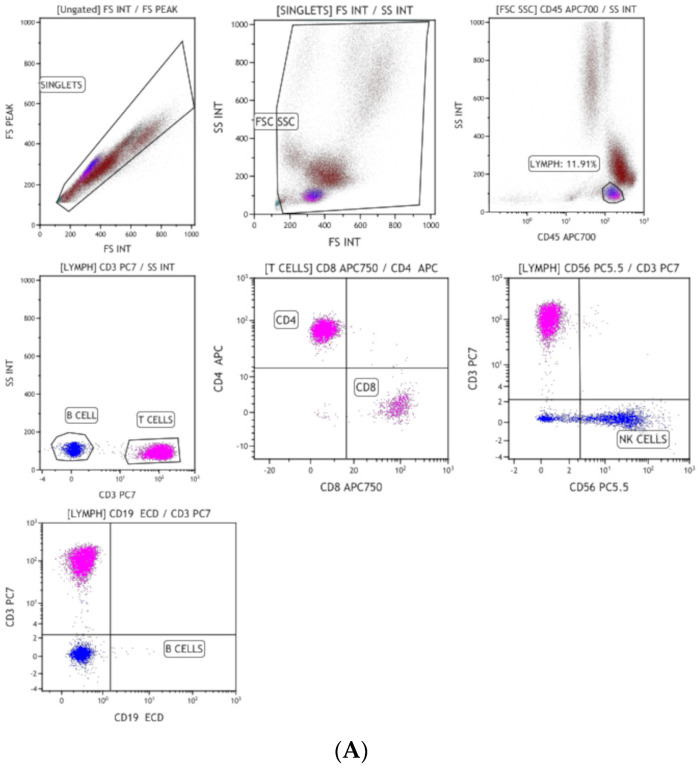

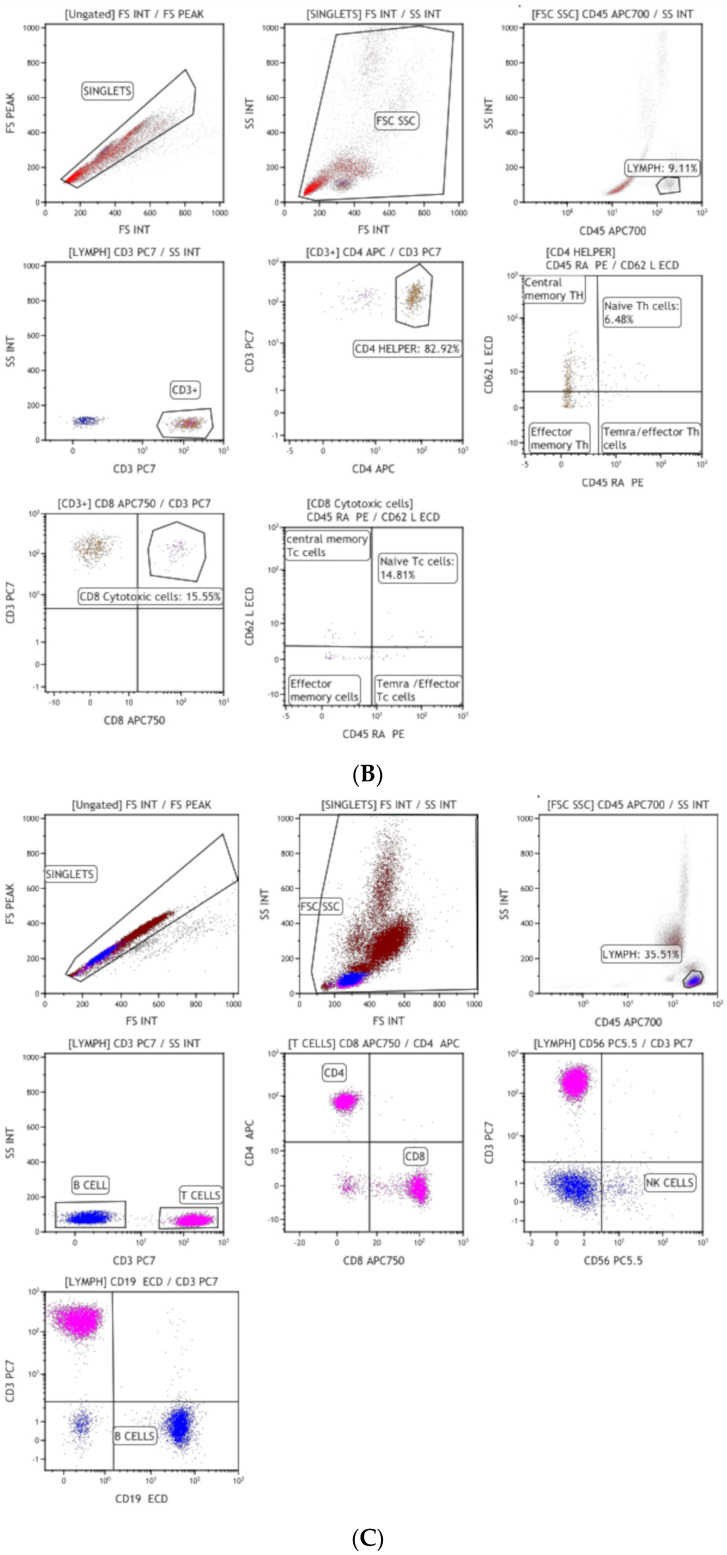

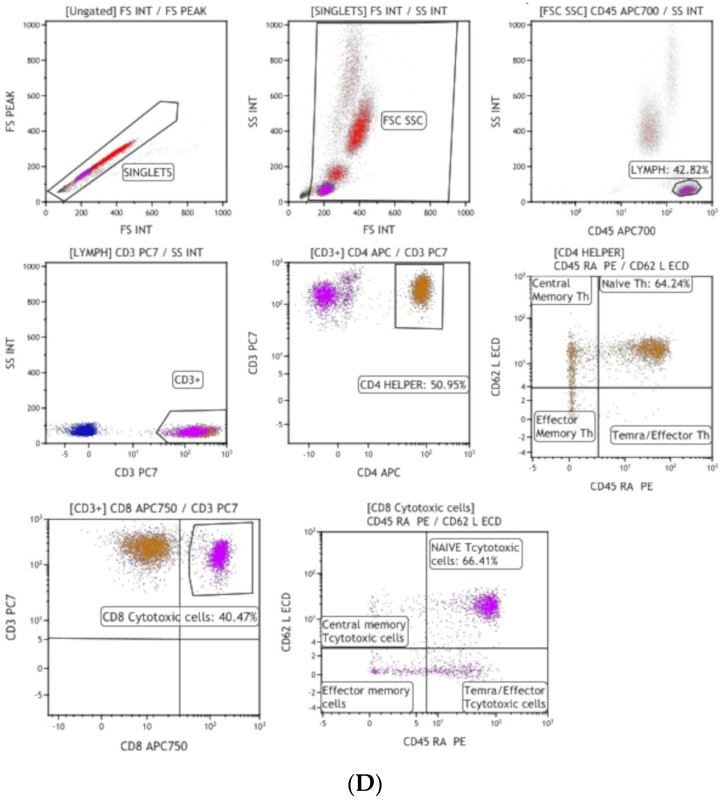
(**A**) Flow cytometry for lymphocyte subsets showing reduced CD3, CD4, and CD8 T cells, absent B cells, and normal NK cells. (**B**) Flow cytometry showing markedly reduced naive helper and cytotoxic T cells, (**C**) lymphocyte subset of a normal control child, and (**D**) naïve helper and cytotoxic T cells in control.

Lymphocyte subset analysis by flow cytometry revealed a deficiency of T cells (CD3–409, Normal 2170-6500; CD4-355, Normal 1580-4850; CD8-56, Normal 680-2470), absent B cells (CD19-0, N 430-3300), and normal NK cells (CD 56-169, Normal 80-340), with deficiencies of naïve helper T cells (CD4/CD45RA/CD62L-154, N-1748-4201) and naïve cytotoxic T cells (CD8/CD45RA/CD62L-71, N 564-1040) (Figure 1). Clinical exome sequencing revealed a homozygous, likely pathogenic, missense variant in exon 2 of the RAG1 gene, resulting in the amino acid substitution of lysine for glutamic acid at codon 770 (c.2308G>A; p. Glu770Lys), confirming the diagnosis of autosomal recessive B negative severe combined immune deficiency [19].

### 3.2. Stool Sample Analysis for Poliovirus Infection

**Figure 2:**
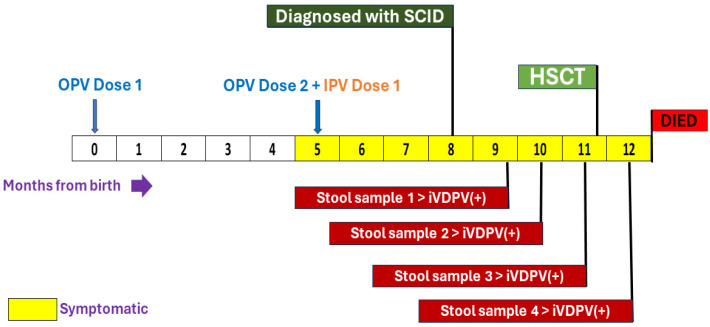
Timeline illustrating polio vaccination, onset of symptoms, excretion of iVDPV, and outcome.

As per the iVDPV Phase I study protocol, his first stool sample was tested for poliovirus infection after the diagnosis of IEI in July 2022, which yielded iVDPV type 1 (16 nucleotide changes from parent Sabin type 1). The second, third, and fourth stool samples collected four weeks apart were also positive for iVDPV type 1 (Figure 2).

### 3.3. Action Taken by the Surveillance Program and Details of the Epidemiological Investigation

As per the country’s emergency preparedness and response plan, on 1 August 2022, information was shared with ICMR Delhi, MOHFW, WHO-SEARO, and WHO HQ. The epidemiological investigation in the community was initiated within 48 h of notification of the VDPV. It started on 2 August 2022 and was completed on 5 August 2022. During this activity, population immunity for polio was assessed, and active case searches in community and health facilities for any missed AFP cases with an onset in last six months and the collection of stool samples from contacts were completed. A total of 89% of children in the age group 24–59 months had three doses of OPV3. Neither the families nor the 33 health facilities visited in and around the community offered a history of any AFP cases during the last six months.

A total of 115 stool samples were collected from the community and were tested for poliovirus. An amount of 114 samples were found to be negative for VDPV. The child’s father, an asymptomatic 32-year-old male, was found to be excreting iVDPV1 with 11 nt changes, but his mother’s stool sample was negative, as well as those of other family members. The father was reported to be closely involved in providing care, including changing diapers, since they had two other young children to look after.

### 3.4. Molecular Characterization of iVDPV by Whole Genome Sequencing

Polioviruses were isolated from four sequentially collected samples using the WHO methodology of the global polio laboratory network for the isolation and identification of poliovirus. iVDPVs isolated from the SCID child and from his father were characterized by Sanger sequencing, which turned out to be iVDPV type 1, with 16, 15, 15, 11, and 11 nucleotides changes, respectively (Table 2). The VP1 region sequencing of his father’s sample was found to be a match with the iVDPV1 excreted by the child, except for a new mutation, T570C. Further genomic level analysis was carried out using Next-Generation Sequencing (NGS).

**Figure 3:**
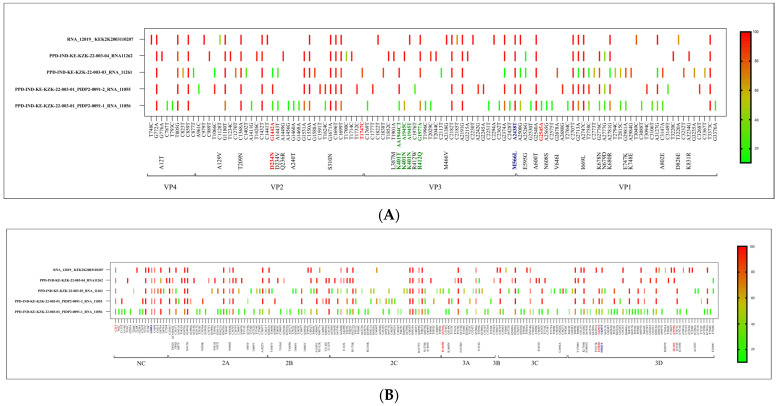
(**A**) All capsid gene (VP4, VP2, VP3, and VP1) figures, with nucleotide/amino acid variants detected in both the proband and the father. The text marked in red are the previously reported neurovirulent mutations, the text marked in blue are nucleotide changes from human poliovirus strain Sabin-1 to the polio 1 wild-type Mahoney strain, and the text marked in green are the mutations detected in the antigenic sites. (**B**) All other gene figures except capsid genes, with nucleotide/amino acid variants detected in both the proband and the father. The text marked in red are the previously reported neurovirulent mutations, and the text marked in blue are nucleotide changes from human poliovirus strain Sabin-1 to the polio 1 wild-type Mahoney strain.

The polio genome consists of non-coding (NC), VP4, VP2, VP3, VP1, 2A, 2B, 2C, 3A, 3B/VPg, 3C, and 3D regions. Nucleotide changes 162, 107, 136, and 102, respectively, were observed in the full genome, whereas 42, 29, 39, and 33 respective amino acid changes were noted. A total of 49 nucleotides and 15 amino acid mutations were identified as common in all four samples of the iVDPV case. Notably, non-synonymous mutations were observed in various genes, specifically in VP2 (N#2), VP3 (N#1), VP1 (N#4), 2A (N#3), 2B (N#2), 2C (N#2), and 3D (N#1) (Table 2, Table 3 and Table S1). Among these, consistent nucleotide changes were found in the NC region (G480A) and VP3 region (A2438T) across all four isolates. Additionally, a variation (C6203T) in the 3D region was observed in the first three samples but not in the fourth sample. These variations were previously associated with neurovirulence [17,18]. Notably, nucleotide changes in the VP3 region (A2438T) and 3D region (C6203T) resulted in amino acid alterations—Met-566-Leu and His-1821-Tyr, respectively (Table 2 and Table 3, Figure 3).

Furthermore, a common nucleotide change (C21T) in the NC region was observed in all samples. In the first three samples, G1442A in the VP2 region led to an amino acid substitution (ASP-234-ASN), and in the 3D gene, the G6202T nucleotide mutation resulted in an amino acid substitution (GLU-1820-ASP). The third sample showed a T1747C nucleotide mutation in the VP2 region. The fourth sample showed A7070G, leading to an amino acid substitution (ILE-2110-VAL) in the 3D region.

### 3.5. Anti-Polio Antibody and Cytokine Production

The results of the micro-neutralization assay revealed that the patient’s serum samples had an antibody titer of 11.31 for poliovirus 1 and 7.11 for poliovirus 3.

The serum sample of the child collected at the hospital for routine tests was used for cytokine analysis. Estimation of cytokine levels in the serum sample collected during the iVDPV excretion revealed higher levels of MCP-1 and MIG and a very low IP-10 level.

### 3.6. Hematopoietic Stem Cell Transplantation

The child underwent a haploidentical hematopoietic stem cell transplantation with TCR alpha beta depleted peripheral blood stem cells from his father. Two days later, he developed a fever and breathlessness, followed by watery diarrhea. His chest X-ray was suggestive of right upper lobe collapse with pneumonitis. Four days post-HSCT, the child’s sensorium worsened, with the progressive deterioration of GCS; his fever persisted, and his breathlessness worsened. He had unilateral LMN facial palsy. He developed cardiogenic shock with echocardiographic features of left ventricular dysfunction and was being mechanically ventilated. His ECG and troponin I levels were suggestive of severe myocarditis. He was engrafted on day +10 and with donor chimerism 92% on day 14. However, despite all efforts, the child expired three weeks following HSCT.

## 4. Discussion

Surveillance among patients with acute flaccid paralysis will not help in the detection of asymptomatic individuals excreting VDPVs, who may be chronic excreters and may result in community spread before they either succumb or develop paralysis. Patients with either IEIs due to B cell defects like X-linked agammaglobulinemia and common variable immune deficiency (CVID) or combined immune deficiency disorders like SCID are at risk of harboring and excreting iVDPVs, and systematic surveillance in this group of patients is the only way to detect the same. Since our patient was a SCID patient, the antibody titer indicates the presence, although low, of antibodies administered passively by IVIg or obtained from the mother’s milk. Individuals with compromised immune systems are particularly vulnerable to infectious agents, and in the context of poliovirus, low antibody levels may lead to a heightened risk of infection and potential disease progression.

A total of 149 iVDPV cases were reported to WHO from January 1961 to December 2019 [20]. The most prevalent serotype was iVDPV type 2 (iVDPV2) (56%), followed by iVDPV type 3 (iVDPV3) (23%) and iVDPV type 1 (iVDPV1) (17%), with 4% heterotypic mixtures. Patients with these inborn errors of immunity, including those with XLA, CVID, and SCID, were usually on prophylaxis with IVIg, and patients with SCID would require an HSCT to prevent mortality. Follow-up visits to the hospital for IVIg at monthly intervals facilitated the collection of stool samples.

In India, the COVID-19 pandemic provided the opportunity to integrate the surveillance of poliovirus excretion among patients with IEIs with AFP surveillance. COVID lockdowns with the resulting disruption of travel also enabled a system to be developed, whereby monthly IVIg prophylaxis was administered at peripheral hospitals [21]. A patient with SCID was identified as a prolonged serotype 3 iVDPV (iVDPV3) excretr and abruptly cleared infection after a period of 2 years [13]. Of the globally reported iVDPV excreters, most stopped excretion within six months or died, but prolonged excretion was reported in at least seven cases [22]. Four countries, namely Egypt, Iran, Jordan, and Tunisia, were selected to pilot the implementation of a system for the surveillance of iVDPV, but implementation was hampered by the COVID-19 pandemic. Further, although the safety of the nOPV2 was established in normal individuals, its safety needs to be established among patients with IEIs [10,23].

RNA viruses incorporate mutations at a higher rate than DNA-containing viruses because of the lack of a proofreading mechanism in RNA replication, and they develop quasi-species when they replicate in the human gut. In the case of IEI patients, the continuous replication of the virus in the gut for a longer duration develops a large number of quasi-species, resulting in virus evolution for fitness. Poliovirus, to adapt to an environment, relies on two different mechanisms, with one being mutation and the other being recombination [24]. There are six attenuating sites recognized in Sabin 1 at nucleotide positions 480G (5′UTR), 935T (VP4), 2438A (VP3), 2795A (VP1), 2879T (VP1), and 6203C (3D), and these are considered neurovirulence sites. Additionally, the mutation at 6203C is also considered responsible for temperature sensitivity, further making the virus fit for survival. We observed a change in G480A at 5′UTR and A2438T in the VP3 region across all four isolates. A mutation (C6203T) in the 3D region responsible for temperature sensitivity was observed in the 3/4 stool sample isolates. The immediate healthy contact of the child, i.e., his father, when tested, was found to be excreting VDPV1. The VP1 region sequencing of this sample was found to be a match with iVDPV1 excreted by the child, except for a new mutation, T570C, which might have emerged during replication in the father’s gut. This further makes a strong case for the spillover of the virus from a case to a close contact, making it important to have IEI surveillance in the community to understand iVDPV transmission.

In our patient, the nucleotide changes and amino acid substitutions observed in the iVDPV1 sequence analysis collectively led to the transition from Sabin 1 to the Mahoney (wild) strain, as reported by Christodoulou in 1990 [17] (Table 2, Table 3 and Table S1, Figure 3). Next-Generation Sequencing (NGS) proved instrumental in the differentiation of vaccine-derived poliovirus type 1 (VDPV1) from wild-type poliovirus. This technology enabled the specific detection of mutations linked to vaccine-derived strains, offering a valuable tool for conducting epidemiological investigations during outbreaks. The high-resolution genomic analysis provided by NGS played a crucial role in assessing the risk of reversion to neurovirulence, as it allowed for the identification of mutations across all relevant genes. This capability enhances our understanding of the potential challenges associated with VDPV1, supporting comprehensive strategies for effective public health responses.

Globally, there are very few reported incidents with evidence of iVDPV transmission to healthy contact cases. Alexander et al. reported VDPV from an unknown source with transmission in an unvaccinated community, wherein 23 children, including a SCID child, were infected [25]. Another report from Spain described the transmission of iVDPV to healthy contacts [26]. Therefore, our report is one of the rare incidents of iVDPV transmission from an IEI child to a healthy contact case in the family. The first iVDPV and cVDPV linkage was reported in the Philippines when there was a large cVDPV2 outbreak in the year 2020. Although there was no direct evidence of the transmission of the VDPV2 detected in an IEI child, the VDPV2 virus isolated from the environmental sample during the outbreak indicated a similar genetic distance from the parental OPV2 strain [18]. 

The capsid inhibitor Pocapavir was administered on a compassionate use basis for several patients excreting iVDPV, with mixed results. Although complete clearing of the virus was observed in some recipients, the rapid emergence of resistance was also observed [27]. Combining Pocapavir with the protease inhibitor V-7404 is expected to avoid the development of antiviral resistance [27]. There are no reports on the prophylactic use of Pocapavir in confirmed cases of inborn errors of immunity before screening for iVDPV. This is due to the risk of acquiring and transmitting antiviral resistance [27,28]. The only feasible option is avoidance of the oral polio vaccine and other live vaccines after neonatal screening for SCID and X–linked agammaglobulinemia by TREC/KREC assay. Hematopoietic stem cell transplantation has been reported to result in the cessation of the excretion of iVDPV following adaptive and innate immune reconstitution [29,30].

Early diagnosis of IEI patients by establishing newborn screening facilities in OPV-using countries, awareness among pediatricians about the consequences of OPV in IEI patients, counseling of parents/caregivers of IEI patients about the contraindication of OPV or any live vaccine and creating a national registry of IEI patients are a few areas to be prioritized for the rational use of OPV to avoid the prolonged excretion of poliovirus.

## 5. Conclusions

GPSAP 2022–2024 envisages a long-term plan for iVDPV surveillance to be developed and integrated with polio surveillance systems to ensure sustainability in the post-polio eradication phase when iVDPVs are likely to be a major threat for the re-emergence of poliovirus.

It is imperative to develop antivirals to poliovirus and to have a strategy in place to treat individuals with inborn errors of immunity who have developed infection with iVDPV. This should go hand in hand with strengthening surveillance among patients with inborn errors of immunity and ensuring global access to newborn screening for these disorders to make polio eradication a reality and to sustain it in the long term. Expansion of IEI surveillance will facilitate early detection and the follow-up of iVDPV excretion to mitigate the risk of iVDPV spread.

## Figures and Tables

**Table 1:** Characteristics of the SCID patient excreting vaccine-derived poliovirus.

Characteristics	VDPV Patient
Age at hospitalization, months	7 months
Sex	Male
Immunodeficiency type	Severe combined immunodeficiency (SCID)
Diagnosis of immunodeficiency (months)	7 months
OPV * doses	2
IPV * dose (age at vaccination in months)	1
Period of poliovirus excretion after 1st detection, months	3 months
Estimated total time of virus excretion at the time of detection, months	3 months
Estimated total time of poliovirus excretion, months	3 months
Maximum nucleotide differences	16
Neutralizing antibody tires §	
Against poliovirus type 1	3.5
Against poliovirus type 3	2.83

* OPV: Oral polio vaccine; IPV: inactivated polio vaccine. §: Reciprocate titers 1:11, 1:7.

**Table 2:** Sequencing results * of the polioviruses isolated from the SCID patient and his father.

Samples	ITD Results	Sequencing Results	Nucleotide Changes
Sample 1	P1 Discordant	iVDPV1 (16 nucleotide)	A21G, T45C, G51T, G61A, C96T, C228T, G234A, A268C, T279C, G297C, A298G, A302G, C489T, A648G, C665T, T894C
Sample 2	P1SL	iVDPV1 (15 nucleotide)	A21G, G61A, A201G, C228T, G234A, A268C, T279C, G297C, A298G, A302G, C405T, T615C, A648G, C789T, T894C
Sample 3	P1SL	iVDPV1 (15 nucleotide)	A21G, G61A, A133G, A201G, C228T, G234A, A268C, T279C, G297C, A298G, A302G, T615C, A648G, C789T, T894C
Sample 4	P1SL	iVDPV1 (11 nucleotide)	G61A, C228T, G234A, A268C, G297C, A298G, A302G, C489T, T741C, C753G, T894C
Contact 1	P1SL	iVDPV1 (11 nucleotide)	A21G, G61A, C228T, G234A, A268C, G297C, A298G, A302G, T570C, A648G, T894C

P1: Poliovirus type 1, P1SL: Sabin-like poliovirus 1, iVDPV1: immunodeficiency-related vaccine-derived poliovirus type 1. * Sanger sequencing.

**Table 3:** Summary of the variant analysis of iVDPV1 virus isolates.

Samples	Sample 1				Sample 2				Sample 3				Sample 4			
	Nucleotide	Amino Acid	Previously Reported Neurovirulent Mutations	Poliovirus Strain Sabin-1 to Mahoney Strain	Nucleotide	Amino Acid	Previously Reported Neurovirulent Mutations	Poliovirus Strain Sabin-1 to Mahoney Strain	Nucleotide	Amino Acid	Previously Reported Neurovirulent Mutations	Poliovirus Strain Sabin-1 to Mahoney Strain	Nucleotide	Amino Acid	Previously Reported Neurovirulent Mutations	Polio-virus Strain Sabin-1 to Mahoney Strain
NC	11	0	G480A	C21T	7	0	G480A	C21T	8	0	G480A	C21T	7	0	G480A	C21T
VP4	5	0			3	0			5	0			4	1		
VP2	15	5		G1442A (AA: D234N)	14	3		G1442A (AA: D234N)	15	4		G1442A (AA: D234N), T1747C	14	3		
VP3	13	4	A2438T (AA:M566L)		9	3	A2438T (AA:M566L)		8	3	A2438T (AA:M566L)		11	4	A2438T (AA:M566L)	
VP1	22	8		G2545A	15	5			21	9			11	7		
2A	18	6			12	7			16	6			14	5		
2B	10	5			6	3			9	4			7	3		
2C	19	5			17	3			19	3			11	3		
3A	7	1			6	1			8	3		A5136G (AA: K1465R)	6	2		
3C	11	1			4	0			11	1			7	1		
3D	31	7	C6203T (AA:H1821Y)	G6202T (AA: E1820D)	14	4	C6203T (AA:H1821Y)	G6202T (AA: E1820D)	16	6	C6203T (AA:H1821Y)	G6202T (AA: E1820D)	10	4		A7070G (AA: I2110V)
	162	42			107	29			136	39			102	33		

## Data Availability

The raw data supporting this article will be made available by the authors without undue reservation.

## Supplementary Materials

The following supporting information can be downloaded at: https://www.mdpi.com/article/10.3390/vaccines12070759/s1, Table S1: Nucleotide and amino acid changes in the retrieved sequences.
