# Supplementary material for: Immunodeficiency-Related Vaccine-Derived Poliovirus (iVDPV) Excretion in an Infant with Severe Combined Immune Deficiency with Spillover to a Parent
Source: Vaccines (Basel). 2024 Jul 9;12(7):759. doi: 10.3390/vaccines12070759 (PMC11281642; doi:10.3390/vaccines12070759)
Supplement: Supplementary file 1 [file vaccines-12-00759-s001.zip › vaccines-2979194-supplementary.pdf]

Table S1. Nucleotide and amino acid changes in the retrieved sequences of two samples

Gene	Nucleotide Nutations	Amino Acid Mutations	Reference Position	Percentage Mutation Frequency			
				PPD-IND-KE-KZK-22-003-01_PIDP2-0091-1_RNA_11056	PPD-IND-KE-KZK-22-003-01_PIDP2-0091-2_RNA_11055	PPD-IND-KE-KZK-22-003-03_RNA_11261	PPD-IND-KE-KZK-22-003-04_RNA11262
NC	C21T		21	99.91	99.87	99.91	99.87
NC	T62C		62	13.89			
NC	C115T		115	29.25	93.50		
NC	A116G		116	17.35			
NC	T191C		191				97.56
NC	G203A		203	22.86			
NC	T206C		206			15.34	
NC	G220A		220	12.60			
NC	C265T		265	31.45	92.64		
NC	T377C		377	99.87	99.84	94.87	92.75
NC	G480A		480	99.54	99.58	91.30	89.87
NC	C599T		599	99.89	99.87	99.62	99.75
NC	C651T		651			78.30	
NC	G670A		670	99.90	99.77	90.12	63.28
NC	G676A		676			16.32	
NC	C726T		726				95.18
VP4	G772A		772	97.42	99.71	98.50	99.36
VP4	G776A	A12T	776				99.60
VP4	C787T		787	21.70			
VP4	T793C		793	20.57			
VP4	T805G		805	99.76	99.74	86.52	85.54
VP4	C823T		823			63.14	
VP4	C859T		859	99.84	99.81	98.60	98.99

VP4	C877T		877			19.43	
VP2	A961C		961		21.77		
VP2	C989T		989				95.09
VP2	T1066C		1066		93.52	10.39	
VP2	G1180T		1180	76.04	99.39	97.83	97.74
VP2	T1234C		1234				97.42
VP2	C1270T		1270			77.23	
VP2	C1368A	T209N	1368	99.81	99.83	95.03	95.85
VP2	C1402T		1402			30.47	
VP2	A1411G		1411	21.55			
VP2	T1426C		1426				95.04
VP2	C1432T		1432	99.10	99.84	97.09	94.47
VP2	G1442A	D234N	1442	23.50	93.42	12.25	
VP2	A1443T	D234V	1443	18.55		24.75	
VP2	A1449G	Q236R	1449				95.08
VP2	A1456G		1456	14.92			
VP2	G1460A	A240T	1460	12.79			
VP2	G1468A		1468	23.05			
VP2	G1531A		1531	76.73	99.85	99.68	99.06
VP2	C1550A		1550	77.77	99.85	98.27	96.39
VP2	G1588A		1588			87.78	
VP2	C1591T		1591		93.44		
VP2	T1624C		1624	22.06	94.00		
VP2	G1671A	S310N	1671	99.88	99.86	97.94	97.98
VP2	C1696T		1696	95.24	99.80	97.34	98.30
VP2	C1699T		1699	76.73	99.79	86.52	98.30
VP2	T1700C		1700				45.00
VP2	T1714C		1714				93.46
VP2	T1732C		1732		94.25		
VP2	T1747C		1747			83.51	

VP3	C1769T		1769	19.11			
VP3	C1777T		1777		93.74		
VP3	C1838T		1838			85.54	
VP3	T1852C		1852				99.22
VP3	T1901A	L387M	1901				98.66
VP3	AA1944CT	K401T	1944	18.08	99.30	96.47	
VP3	A1945C	K401N	1945	59.80			98.13
VP3	C1976T	R412W	1976		25.96		
VP3	G1977A	R412Q	1977	69.95		88.89	98.10
VP3	T1996C		1996	11.57			
VP3	T2029C		2029	19.79			90.91
VP3	T2044C		2044				98.59
VP3	C2113T		2113			10.96	
VP3	C2182T		2182	95.20	99.51	97.86	97.56
VP3	A2191G		2191	99.81	99.75	99.58	99.88
VP3	G2215A		2215				97.07
VP3	A2242G		2242		93.08		
VP3	G2245A		2245		92.97		
VP3	C2251T		2251	11.85			
VP3	C2362T		2362	15.76			
VP3	G2374A		2374	74.95	93.69	11.97	95.79
VP3	C2428T		2428	11.48			
VP3	A2438T	M566L	2438	99.76	99.81	97.16	96.47
VP1	A2500G		2500	49.54	94.34	15.79	
VP1	A2526G	E595G	2526			14.63	12.74
VP1	G2530T		2530	11.80			
VP1	G2540A	A600T	2540	99.65	99.70	98.88	98.21
VP1	G2545A		2545	12.52			
VP1	A2565G	N608S	2565	11.74			
VP1	C2575T		2575	14.91			

VP1	G2678A	V646I	2678			84.11	
VP1	A2680G		2680		91.38		
VP1	T2704C		2704	10.92			
VP1	C2707T		2707	99.51	99.79	96.77	97.04
VP1	G2713A		2713	73.32	93.29	17.09	97.89
VP1	A2747C	I669L	2747	99.87	99.82	97.81	98.43
VP1	T2758C		2758	40.44	93.71	14.77	
VP1	C2773T		2773			60.23	
VP1	G2776C	K678N	2776	29.36	91.98	81.25	98.23
VP1	A2777G	N679D	2777	21.00	45.00		38.00
VP1	A2781G	K680R	2781	71.25	92.81	10.61	97.55
VP1	T2794C		2794			55.90	
VP1	T2815C		2815	24.86		57.06	
VP1	G2981A	E747K	2981	24.36		84.78	
VP1	A2984G	K748E	2984			85.40	
VP1	C3088T		3088	28.21			
VP1	T3094C		3094		91.93		
VP1	C3106T		3106	28.10			
VP1	A3127G		3127	37.53	93.95	11.11	
VP1	C3147A	A802E	3147	25.64		86.36	
VP1	C3149T		3149		25.42		
VP1	T3220C		3220				98.49
VP1	C3232T		3232			82.91	
VP1	A3234G	K831R	3234				96.52
VP1	G3235A		3235			66.46	
VP1	C3268T		3268		90.54		
VP1	C3367T		3367			61.89	
VP1	T3373C		3373	99.29	99.85	97.24	97.81
VP1	G3376A		3376	21.88			
2A	C3391T		3391	75.37	99.80	98.07	98.25

2A	AC3416GT	T892V	3416			78.04	98.21
2A	C3417T	T892I	3417	75.48	90.68	19.26	
2A	A3431G	I897V	3431		22.53		
2A	T3433A		3433				98.57
2A	G3478A		3478	74.82	99.81	97.47	97.87
2A	A3491G	N917D	3491	99.80	99.78	100.00	99.26
2A	A3496G		3496		90.67		
2A	A3514G		3514				99.13
2A	A3517G		3517	26.32			
2A	T3535C		3535	25.89			
2A	C3559A	N939K	3559		87.33		
2A	A3562G		3562	24.34			
2A	C3571T		3571				94.18
2A	G3589A		3589			65.88	
2A	T3593C	Y951H	3593				98.33
2A	G3611A	V957I	3611	11.06		83.33	
2A	G3622A		3622	23.53			
2A	G3628A		3628	75.78	99.93	93.01	93.81
2A	T3643C		3643	22.37			
2A	A3644G	N968D	3644	76.43	99.92	99.42	99.30
2A	C3670T		3670	76.22	99.73	97.02	97.30
2A	C3679T		3679				95.15
2A	C3694T		3694	16.83		59.19	
2A	A3697G		3697				99.14
2A	T3715C		3715	23.39			
2A	A3719G	I993V	3719		25.13		
2A	T3730C		3730			10.60	
2A	A3752G	I1004V	3752	24.33			
2A	T3754C		3754			55.22	
2A	C3790T		3790	33.57		16.92	

2A	C3807T	A1022V	3807	46.20	99.88	97.23	98.61
2A	C3811T		3811			17.44	
2B	G3862A		3862	58.89		86.40	96.39
2B	T3869G	F1043V	3869			10.37	
2B	A3880G		3880		93.12		
2B	T3883C		3883				98.51
2B	C3909T	T1056I	3909	21.97			
2B	A3910G		3910				91.04
2B	G3916A		3916	13.22			
2B	A3917T	T1059S	3917	23.26			
2B	T3922C		3922			13.65	
2B	A3938G	I1066V	3938			19.08	
2B	T3955C		3955	23.51			
2B	C3967T		3967	39.91	93.59	74.13	
2B	A3992G	I1084V	3992	15.71			
2B	C4012T		4012	58.47		24.11	94.40
2B	T4045C		4045			20.79	
2B	C4056T	A1105V	4056	60.95	99.80	95.00	94.76
2B	C4090T		4090		93.27		
2B	G4094A	V1118I	4094		91.73		99.55
2B	C4116T	T1125I	4116	98.56	99.91	96.57	96.19
2C	A4156G		4156			10.28	
2C	A4165G		4165				96.50
2C	A4174G		4174	10.52	91.00	58.82	
2C	A4204G		4204	83.59		25.25	99.30
2C	A4229C	I1163L	4229				97.71
2C	T4243C		4243	57.46	74.13	33.52	97.09
2C	T4261C		4261	11.95			98.10
2C	G4275A	R1178K	4275	67.46	99.91	95.49	96.24
2C	T4357C		4357		24.43	67.11	

2C	T4360C		4360	22.71			
2C	A4372G		4372			26.79	
2C	G4387A		4387	13.87			
2C	G4389A	R1216K	4389	28.41			
2C	G4411A		4411		25.57	57.85	
2C	G4429A		4429			38.33	
2C	C4499T		4499		68.90		
2C	A4504G		4504		26.60		
2C	C4510T		4510	25.78			
2C	A4567G		4567		65.01		
2C	A4573G		4573	10.13	27.02	55.81	
2C	C4624T		4624		67.05		
2C	G4642A		4642		23.33		
2C	C4654T		4654	28.10			
2C	T4675C		4675	18.87			
2C	G4699A		4699			50.88	
2C	A4750G		4750			52.67	
2C	C4810T		4810	79.98	99.76	95.04	92.68
2C	G4819A		4819	94.95	99.85	97.87	97.87
2C	C4855T		4855	13.48			
2C	A4859G	M1373V	4859	75.21	30.52	96.67	99.86
2C	T4867A		4867	76.40	99.69	98.75	98.26
2C	A4878G	N1379S	4878	39.93	23.70	34.83	
2C	G4949T	A1403S	4949	10.64			
2C	T4984C		4984				95.91
2C	T5032C		5032		66.04		
2C	C5080T		5080			18.04	
2C	T5092C		5092			54.01	
3A	A5136G	K1465R	5136			17.35	
3A	C5146T		5146	12.59			

3A	G5149C	K1469N	5149	15.12	90.83	25.71	
3A	T5155C		5155		20.98		
3A	T5161C		5161				87.83
3A	T5170C		5170	81.39	99.58	97.11	96.59
3A	A5174G	N1478D	5174				100.00
3A	T5180C		5180			55.17	
3A	G5206A		5206	26.53			
3A	T5233C		5233		58.51		
3A	C5242T		5242			15.68	
3A	A5266G		5266	57.72	99.69	97.59	97.67
3A	T5295G	I1518S	5295			11.01	10.00
3A	G5308A		5308			61.33	
3A	C5314T		5314	31.24			
3A	C5320A		5320	26.17			
3A	T5350C		5350		27.66		
3A	C5354T		5354				94.11
3C	C5479T		5479	64.86	99.88	97.00	96.54
3C	C5500T		5500	18.39			
3C	A5572G		5572			30.57	
3C	G5608A		5608				98.87
3C	G5620A		5620	70.70	99.87	98.55	99.24
3C	C5623T		5623			15.98	
3C	A5626G		5626	17.08			
3C	T5656C		5656	21.88			
3C	A5674G		5674	70.03	99.83	99.21	98.83
3C	C5692T		5692	13.06			
3C	A5696C		5696				96.57
3C	C5699T	P1653S	5699	27.18			97.13
3C	A5707C		5707	10.35	97.64	91.19	
3C	T5710C		5710				97.37

3C	C5758T		5758	24.26			
3C	T5779A		5779			17.55	
3C	T5809C		5809			20.31	
3C	C5810T		5810	23.48			
3C	G5823C	G1694A	5823			11.54	
3C	C5827T		5827			91.67	
3C	T5926C		5926			10.05	
3D	T6010C		6010	13.26			
3D	C6034T		6034			47.10	
3D	T6070C		6070	67.90	99.83	97.26	97.16
3D	T6080C	Y1780H	6080				92.86
3D	C6115T		6115	30.99			
3D	A6120G	K1793R	6120	68.37	99.92	98.75	98.56
3D	A6143G	N1801D	6143	13.75			
3D	A6157G		6157	13.52			
3D	G6169A		6169		68.14		
3D	G6191A	E1817K	6191	55.08			
3D	G6202T	E1820D	6202	11.05	97.16	91.35	
3D	C6203T	H1821Y	6203	53.88	70.88	65.88	
3D	A6211G		6211			11.78	
3D	C6248T		6248	23.62			
3D	A6262G		6262	22.32			
3D	T6316C		6316	12.09	97.63	85.44	
3D	G6322A		6322	11.51			
3D	C6328T		6328		26.33		
3D	C6424T		6424	59.30			97.81
3D	G6448A		6448	21.92			
3D	T6457C		6457	10.62	97.21	94.87	
3D	A6517G		6517	28.11			
3D	T6556C		6556	13.64			

3D	T6580C		6580	11.01	97.57	95.98	
3D	A6655G		6655	44.35			100.00
3D	T6656C		6656	10.02	97.36	95.57	
3D	C6724T		6724	23.94			
3D	C6766T		6766				97.61
3D	C6781T		6781	15.84			
3D	G6814A		6814	54.54	99.83	89.34	91.02
3D	A6997G		6997	16.56			
3D	T7045C		7045				94.12
3D	A7070G	I2110V	7070				99.41
3D	T7071C	I2110T	7071	18.43	97.40	96.77	
3D	A7072G	I2110M	7072	16.91			
3D	C7213T		7213	23.13			
3D	G7286A	A2182T	7286			15.77	
3D	A7321G		7321			16.32	
3D	T7322C		7322		23.20		
3D	A7339G		7339	14.98			
3D	G7345A		7345	45.81	97.62	88.61	
3D	T7360C		7360	17.76			
3D	T7368G	F2209C	7368			11.43	11.67
**Total Nuc mutations**				162	107	136	102
**Total AA mutations**				42	29	39	33
